# New Methods of Teaching and Learning in Libraries

**DOI:** 10.5195/jmla.2018.418

**Published:** 2018-04-01

**Authors:** Margaret A. Hoogland

**Affiliations:** Mulford Health Sciences Library, University of Toledo, Toledo, OH

*New Methods of Teaching and Learning in Libraries* by Ann Whitney Gleason is the eleventh book in a series discussing a range of topics that are important to health sciences librarianship. Rowman & Littlefield, in conjunction with the Medical Library Association, is publishing and promoting this book series. Each book covers a different and pertinent aspect of librarianship, and Gleason chooses to address the need for reexamining how librarians and patrons are teaching and learning.

The book consists of three distinct parts: “Teaching and Learning Practices in Library Instruction,” “Using Educational Technology to Scaffold Learning,” and “Facilitating Learning in Library Spaces.” This arrangement gives the reader the option of focusing on the chapters of particular relevance or reading the entire book.

For a student enrolled in a master’s in library science program, “Teaching and Learning Practices in Library Instruction” provides a history of teaching in library science programs and emphasizes the need to adapt courses to meet the challenges that new graduates will face in the workplace. This book does a great job of identifying and providing solutions, which makes this text potentially useful as a core or recommended book in library science programs.

Mid-career professionals will find “Educational Technology to Scaffold Learning” relevant, especially if they are taking on additional teaching responsibilities. Additionally, this section would be useful for librarians who are considering new ways to use videos, learning management systems, or other technology in a course.

Directors, new professionals, and future planners of libraries will find “Facilitating Learning in Library Spaces” beneficial. This section addresses ways to reallocate current spaces and provides suggestions for libraries that are choosing to implement a patron-centered outlook. Although some reorganization and rethinking of services is necessary, this approach provides numerous opportunities to increase existing patrons’ use of the library and extend services to new community members.

*New Methods of Teaching and Learning in Libraries* makes reading about the latest trends in library science enjoyable, and every professional, new graduate of a library science program, or new hire could benefit from reading this book.

